# Association between the systemic inflammation response index and iron deficiency anemia in US adults, 2003–2018: A population-based cross-sectional analysis

**DOI:** 10.1097/MD.0000000000047302

**Published:** 2026-01-23

**Authors:** Yang Li, Tong Li

**Affiliations:** a Department of Hematology, The First Hospital of China Medical University, Shenyang, China; b Department of Oncology Medicine, The First Hospital of China Medical University, Shenyang, China.

**Keywords:** biomarker, C-reactive protein, iron deficiency anemia, multiple imputation, nonlinear association, restricted cubic splines, systemic inflammation response index

## Abstract

Systemic inflammation shapes iron homeostasis and can contribute to functional iron deficiency. The systemic inflammation response index (SIRI) – computed from neutrophil, monocyte, and lymphocyte counts – captures the balance between pro-inflammatory and immune-regulatory pathways. Its population-level relationship with iron deficiency anemia (IDA), including potential nonlinearity, is not well characterized. This study aims to examine the association between SIRI and prevalent IDA in US adults using the National Health and Nutrition Examination Survey (NHANES), with a priori interest in nonlinearity. We conducted a cross-sectional analysis of 11,047 adults from NHANES 2003 to 2010 and 2015 to 2018. SIRI was calculated as (Neutrophils × Monocytes)/Lymphocytes and analyzed as lnSIRI and by tertiles. IDA was defined using established hemoglobin and iron-status criteria. Survey-weighted multivariable logistic regression estimated odds ratios (ORs) and 95% confidence intervals (CIs), with multiple imputation for missing data. Restricted cubic splines (3 knots at the weighted 10, 50, and 90 percentiles of raw SIRI) assessed nonlinearity. Sensitivity analyses excluded participants with C-reactive protein (CRP) > 10 mg/L; severity analyses distinguished mild versus moderate-to-severe IDA. In the fully adjusted model, lnSIRI was positively associated with IDA (OR: 1.38; 95% CI: 1.11–1.72; *P* = .004). Compared with the lowest tertile, the highest SIRI tertile had higher odds of IDA (OR: 1.55; 95% CI: 1.22–1.97; *P* for trend < .001). The exposure–response curve was nonlinear (*P* for nonlinearity = .012), with a threshold around SIRI ≈ 0.60 (*P* for nonlinearity = .012), below which the association was inverse and above which it was positive. Findings were robust after excluding high-CRP individuals and were more pronounced for moderate-to-severe IDA (OR for lnSIRI 1.48; 95% CI: 1.10–2.00), whereas mild IDA showed weaker associations. In this large, nationally representative NHANES sample, higher SIRI is independently associated with the prevalence of IDA, following a nonlinear pattern with a threshold near 0.6. SIRI may serve as an accessible biomarker to help flag individuals at risk of inflammation-related iron deficiency; causal inference is limited by the cross-sectional design.

**Key Points**

- Nationally representative US adults (n = 11,047; NHANES 2003–2010, 2015–2018).
- Higher SIRI is associated with greater odds of IDA.
- The association is nonlinear with a threshold around SIRI ≈ 0.60.
- Survey-weighted, multiply imputed logistic models with restricted cubic splines.
- Findings robust after excluding CRP > 10 mg/L and stronger for moderate-to-severe IDA.

## 1. Introduction

Iron deficiency anemia (IDA) is the most common nutritional disorder worldwide, representing a significant global public health challenge that affects quality of life, impairs cognitive function, and reduces work productivity.^[1-3]^ While often associated with inadequate dietary intake or chronic blood loss, a substantial proportion of anemia cases, particularly in clinical settings, are linked to underlying inflammatory states.^[4-6]^ This condition, termed anemia of inflammation or anemia of chronic disease, is recognized as the second most prevalent form of anemia globally and is common among hospitalized or chronically ill patients.^[7,8]^

The pathophysiological link between inflammation and iron metabolism is well-established and pivots on the hormone hepcidin, the master regulator of systemic iron homeostasis.^[9,10]^ During periods of prolonged immune activation, such as in chronic infections, autoimmune diseases, or cancer, pro-inflammatory cytokines, most notably Interleukin-6, stimulate hepatic hepcidin synthesis.^[11-13]^ Elevated circulating hepcidin then binds to and induces the degradation of ferroportin, the sole known cellular iron exporter protein. This action has a dual effect: it blocks the absorption of dietary iron from duodenal enterocytes and sequesters iron recycled from senescent erythrocytes within macrophages of the reticuloendothelial system.^[14,15]^ The resulting hypoferremia leads to a state of functional iron deficiency, where iron is abundant within the body but unavailable for erythropoiesis, ultimately culminating in iron-restricted red blood cell production and anemia.^[16-18]^ At the population level, more pro-inflammatory diets – as reflected by higher dietary inflammatory index scores – are also associated with higher anemia prevalence in US adults, further supporting an inflammation–iron axis.^[19]^

Given the central role of inflammation in iron dysregulation, accurate and accessible biomarkers of systemic inflammatory status are crucial for both clinical and epidemiological research. The systemic inflammation response index (SIRI) has emerged as a novel, integrated biomarker calculated from routine complete blood counts.^[20,21]^ Defined by the formula (Neutrophil count × Monocyte count)/Lymphocyte count, SIRI captures the dynamic interplay between pro-inflammatory myeloid cells (neutrophils and monocytes) and the adaptive immune system’s regulatory cells (lymphocytes).^[22]^ Neutrophils and monocytes are key effectors in the innate immune response and are involved in cytokine production and iron sequestration, while lymphocytes play a crucial role in modulating inflammation.^[23]^ The utility of SIRI as a prognostic marker has been demonstrated across a spectrum of conditions, including cardiovascular diseases,^[24]^ autoimmune disorders,^[21]^ and various malignancies,^[25,26]^ validating its capacity to reflect the systemic inflammatory burden.

Despite growing evidence for the utility of composite inflammatory indices, the specific association between SIRI and IDA remains largely unexplored. While a related marker – the systemic immune-inflammation index (SII) – has already been associated with anemia in US adults and in US children, and even with iron deficiency and iron-deficiency anemia via iron-biomarker profiles, these studies did not directly evaluate SIRI against IDA; hence a focused investigation is warranted.^[27-29]^ Furthermore, the nature of this relationship – whether it is linear or exhibits a threshold effect – is unknown. A nonlinear association could suggest that the risk of IDA only escalates after a certain threshold of inflammation is surpassed, a finding with potential clinical relevance for risk stratification. Therefore, this study aimed to examine the association between SIRI and the prevalence of IDA using data from a large, nationally representative sample of US adults from the National Health and Nutrition Examination Survey (NHANES).

## 2. Materials and methods

### 2.1. Data source and study population

This study used publicly available data from NHANES, a continuous, cross-sectional program conducted by the National Center for Health Statistics to assess the health and nutritional status of the civilian, noninstitutionalized US population. NHANES employs a complex, multistage, stratified probability sampling design to provide nationally representative estimates. Because the relevant variables were unavailable in 2011 to 2014, we pooled 6 cycles for analysis: 2003 to 2004, 2005 to 2006, 2007 to 2008, 2009 to 2010, 2015 to 2016, and 2017 to 2018.

**Figure 1.**
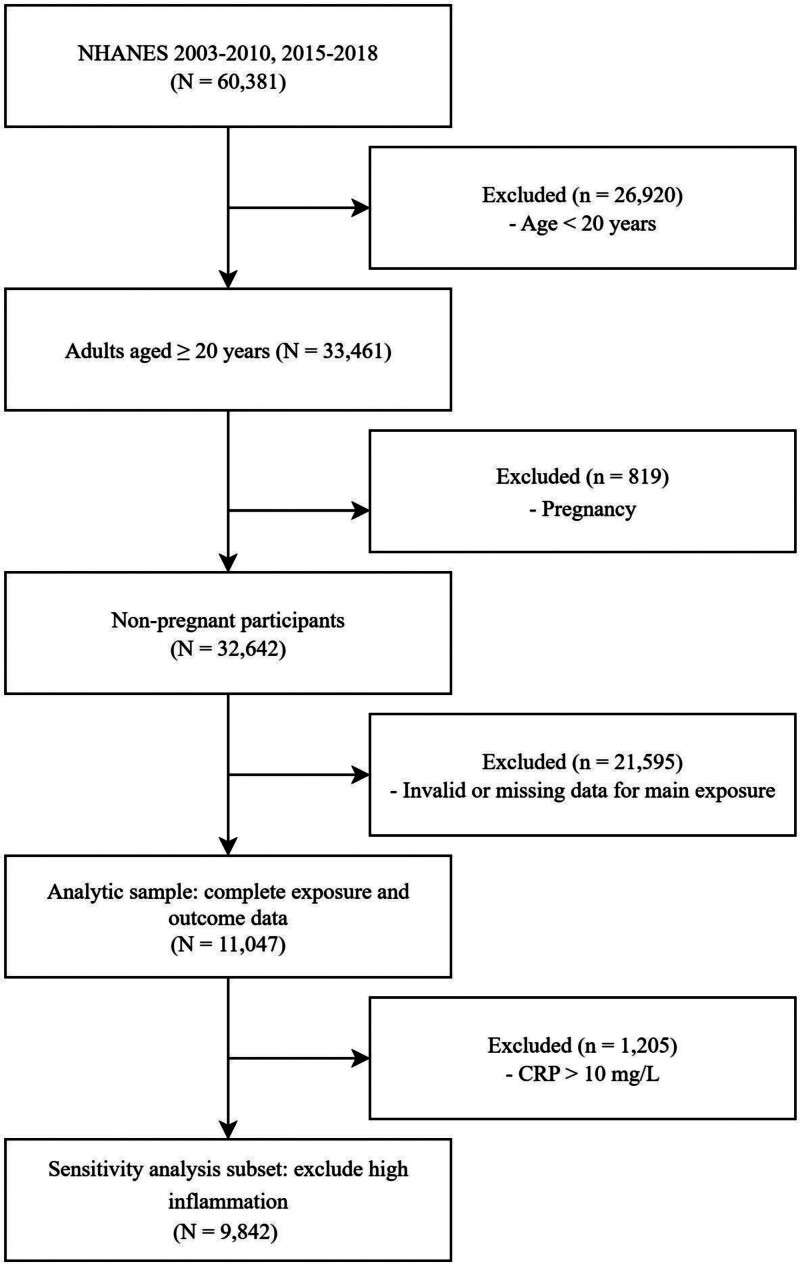
Flowchart of participant selection from the National Health and Nutrition Examination Survey (NHANES) 2003 to 2010 and 2015 to 2018. NHANES = National Health and Nutrition Examination Survey.

Inclusion criteria for this study were adults aged ≥20 years. We excluded participants who were pregnant or had missing data on the main exposure (SIRI components) or outcome (IDA status) variables. For a prespecified sensitivity analysis, we further excluded participants with C-reactive protein (CRP) > 10 mg/L, a marker of acute or high-grade inflammation. A detailed flowchart of the participant selection process is provided in Figure 1. The NHANES protocol was approved by the National Center For Health Statistics Institutional Review Board, and all participants provided written informed consent.

### 2.2. Exposure variable: SIRI

Differential blood cell counts, including neutrophils, monocytes, and lymphocytes, were measured as part of the complete blood count using automated hematology analyzers in the NHANES mobile examination centers. The SIRI was calculated using the following formula:


$$\begin{array}{l} SIRI=\frac{Neutrophil\;\;\;count\left(\times 10^{3}/\;\mu \;L\right)\times Monocyte\;\;\;count\left(\times 10^{3}/\;\mu \;L\right)\;\;\;}{Lymphocyte\;\;\;count\left(\times 10^{3}/\;\mu \;L\right)} \end{array}$$

Due to its right-skewed distribution, SIRI was natural log-transformed (lnSIRI) to approximate normality for inclusion as a continuous variable in regression models. For categorical analyses, SIRI was divided into tertiles based on its distribution in the weighted study population.

### 2.3. Outcome variable: IDA

The primary outcome was IDA, defined by the concurrent presence of anemia and iron deficiency. Anemia followed World Health Organization criteria: hemoglobin < 12.0 g/dL for nonpregnant females and <13.0 g/dL for males. Iron deficiency was defined as ferritin < 25 µg/L, a threshold supported by recent evidence as physiologically aligned with the onset of iron-restricted erythropoiesis in populations with prevalent low-grade inflammation. This cutoff is increasingly recognized as more specific than the traditional <15 µg/L threshold in settings where inflammation can falsely elevate ferritin levels. In a secondary severity analysis, mild IDA was defined as hemoglobin 11.0 to 11.9 g/dL in females and 11.0 to 12.9 g/dL in males, whereas moderate-to-severe IDA was defined as hemoglobin < 11.0 g/dL in both sexes.

### 2.4. Covariates

**Figure 2.**
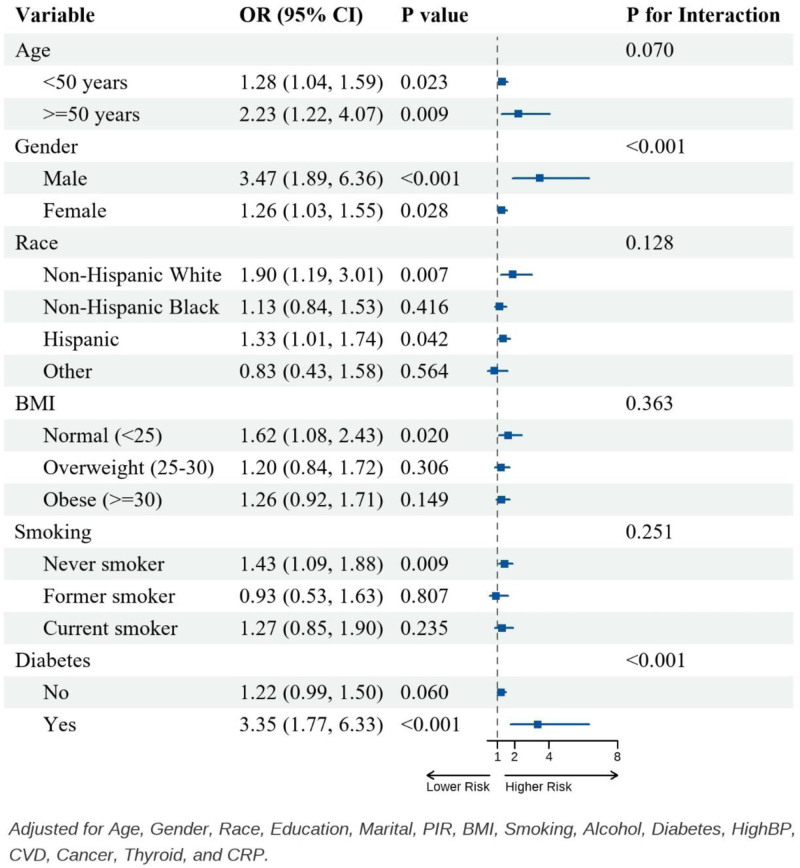
Subgroup analysis of the association between SIRI and IDA. IDA = iron deficiency anemia, SIRI = systemic inflammation response index.

We selected covariates a priori on causal grounds to reduce confounding rather than to optimize model fit. Models adjusted for demographic structure (age, sex, race/ethnicity), socioeconomic position (education, poverty–income ratio, marital status), lifestyle factors (smoking, alcohol use), adiposity (body mass index), and chronic comorbidities that plausibly influence both systemic inflammation and iron biology (diabetes, hypertension, cardiovascular disease, cancer, thyroid disease). CRP was handled in 2 ways: in the direct-effect specification we included CRP to estimate the association of SIRI with IDA independent of generalized acute-phase inflammation, and in sensitivity analyses we restricted to CRP ≤ 10 mg/L to mitigate ferritin misclassification during acute/high-grade inflammation. To avoid overadjustment and collinearity with the exposure, we did not adjust for total white blood cell count or for biomarkers used to define IDA. Full variable definitions and codings are provided in the legend for Figure 2 and in the Supplementary materials referenced below.

### 2.5. Statistical analysis

All analyses were performed in R (version 4.5.1) and accounted for NHANES’ complex design using strata, primary sampling units, and examination weights. For pooled analyses across 6 2-year cycles, we derived combined examination weights by dividing the 2-year MEC weights by the number of included cycles. Missing covariate data were addressed with multiple imputation by chained equations (*m* = 5), and estimates were combined using Rubin’s rules. Descriptive characteristics are reported as survey-weighted means and standard deviations for continuous variables and survey-weighted percentages for categorical variables; differences by IDA status were assessed with design-based *t* tests for continuous variables and Rao–Scott chi-square tests for categorical variables. Associations between SIRI and IDA were evaluated with multivariable logistic regression, estimating odds ratios (ORs) and 95% confidence intervals (CIs) for lnSIRI and for SIRI tertiles. We fit a sequence of models: an unadjusted model; a model adjusted for age, sex, and race/ethnicity; a model additionally adjusting for education, marital status, poverty-to-income ratio, body mass index, smoking, alcohol use, and comorbidities (total-effect specification); and a final model further adjusting for CRP (direct-effect specification to evaluate the association independent of CRP-indexed inflammation). Dose–response was examined using restricted cubic splines with 3 knots at the survey-weighted 10, 50, and 90 percentiles of raw SIRI; nonlinearity was tested by a likelihood ratio comparison of the spline model with its linear counterpart. Subgroup analyses used the fully adjusted, CRP-adjusted model and were stratified by age (<50 vs ≥50 years), sex, race/ethnicity, body mass index (<25 vs ≥25 kg/m²), smoking status, and diabetes; interaction *P*-values were obtained from multiplicative interaction terms with lnSIRI. Sensitivity analyses repeated primary and spline models after excluding participants with CRP > 10 mg/L. Severity analyses used separate multinomial or binary logistic regressions to compare mild and moderate-to-severe IDA, respectively, against the non-IDA reference. Two-sided *P* < .05 was considered statistically significant.

## 3. Results

### 3.1. Baseline characteristics of the study population

The participant selection process is detailed in Figure 1. Of the 60,381 participants initially identified in the selected NHANES cycles, we excluded 26,920 individuals younger than 20 years and 819 pregnant individuals. After further excluding 21,595 participants with missing data for exposure or outcome variables, the final analytic sample for this study consisted of 11,047 adults. For the sensitivity analysis, an additional 1205 participants with CRP levels > 10 mg/L were excluded, resulting in a final sensitivity cohort of 9842 individuals.

The baseline characteristics of the 11,047 participants, stratified by IDA status, are presented in Table 1. The overall prevalence of IDA in the study cohort was 6.4%. Compared with non-IDA participants, those with IDA were younger (mean 38.9 vs 41.3 years, *P* = .047) and predominantly female (95.3% vs 77.5%, *P* < .001). The distribution of race/ethnicity differed markedly across IDA status (*P* < .001): non-Hispanic Black and Hispanic participants were overrepresented among IDA cases, whereas non-Hispanic White participants were under-represented. Socioeconomic position was lower in the IDA group (poverty-to-income ratio, *P* < .001). Never-smoking and nondrinking were more common in IDA, whereas major comorbidities showed small or no absolute differences at baseline. As expected by definition, hemoglobin and ferritin were substantially lower in IDA (both *P* < .001). Unadjusted median SIRI did not differ by IDA status (*P* = .60), indicating that crude SIRI alone is insufficient to discriminate IDA without covariate adjustment.

**Table 1:** Baseline characteristics of the study population by IDA status (full cohort).

Characteristic	Overall, N = 11,047*	No IDA, N = 10,344*	IDA, N = 703*	*P*-value†
Age, yr	41.2 (14.8)	41.3 (14.9)	38.9 (11.1)	.047
Sex				<.001
Male	2395.0 (21.6%)	2361.0 (22.5%)	34.0 (4.7%)	
Female	8652.0 (78.4%)	7983.0 (77.5%)	669.0 (95.3%)	
Race/ethnicity				<.001
Non-Hispanic White	4227.0 (63.4%)	4098.0 (64.7%)	129.0 (39.1%)	
Non-Hispanic Black	2433.0 (12.1%)	2147.0 (11.1%)	286.0 (31.3%)	
Hispanic	2973.0 (15.8%)	2748.0 (15.5%)	225.0 (21.8%)	
Other	1414.0 (8.7%)	1351.0 (8.8%)	63.0 (7.7%)	
Education				.002
High school	2448.0 (23.4%)	2279.0 (23.3%)	169.0 (24.4%)	
<High school	2331.0 (13.4%)	2154.0 (13.1%)	177.0 (19.0%)	
>High school	6268.0 (63.2%)	5911.0 (63.6%)	357.0 (56.5%)	
Marital status				.8
Married/living with partner	6509.0 (62.7%)	6099.0 (62.7%)	410.0 (62.8%)	
Never married	2474.0 (21.3%)	2298.0 (21.3%)	176.0 (22.2%)	
Divorced/separated/widowed	2064.0 (15.9%)	1947.0 (16.0%)	117.0 (15.0%)	
Poverty-to-income ratio	2.9 (1.7)	2.9 (1.7)	2.5 (1.6)	<.001
Smoking status				<.001
Never	6796.0 (59.4%)	6285.0 (58.7%)	511.0 (73.1%)	
Former	1937.0 (19.4%)	1855.0 (19.8%)	82.0 (11.1%)	
Current	2314.0 (21.1%)	2204.0 (21.4%)	110.0 (15.8%)	
Alcohol use				<.001
Nondrinker	4314.0 (32.4%)	3983.0 (31.9%)	331.0 (42.2%)	
Drinker	6733.0 (67.6%)	6361.0 (68.1%)	372.0 (57.8%)	
Body mass index, kg/m²	29.0 (7.5)	29.0 (7.5)	29.8 (8.0)	.072
Diabetes				.8
Yes	1062.0 (7.2%)	1004.0 (7.2%)	58.0 (8.3%)	
No	9762.0 (91.1%)	9129.0 (91.1%)	633.0 (89.8%)	
Borderline	215.0 (1.6%)	203.0 (1.6%)	12.0 (1.9%)	
Hypertension				.6
Yes	2891.0 (22.9%)	2732.0 (22.9%)	159.0 (21.9%)	
No	8136.0 (77.0%)	7592.0 (76.9%)	544.0 (78.1%)	
Cardiovascular disease	534.0 (3.8%)	512.0 (3.8%)	22.0 (2.7%)	.2
Cancer	763.0 (7.5%)	734.0 (7.6%)	29.0 (4.2%)	.019
Thyroid disease	1127.0 (11.0%)	1060.0 (11.1%)	67.0 (9.6%)	.5
White blood cell count, 10³ cells/µL	7.4 (3.0)	7.5 (3.1)	7.1 (2.2)	.003
C-reactive protein, mg/L	4.2 (7.3)	4.2 (7.4)	3.9 (5.3)	.6
Hemoglobin, g/dL	13.8 (1.4)	13.9 (1.2)	10.7 (1.1)	<.001
Ferritin, ng/mL	99.5 (131.7)	104.2 (133.4)	9.0 (5.4)	<.001
Anemia (component)	1239.0 (7.9%)	536.0 (3.1%)	703.0 (100.0%)	<.001
Iron deficiency (component)	2315.0 (19.3%)	1612.0 (15.1%)	703.0 (100.0%)	<.001
SIRI	1.2 (0.8)	1.2 (0.8)	1.2 (0.8)	.6
ln(SIRI)	0.0 (0.6)	0.0 (0.6)	0.0 (0.7)	.6

IDA = iron deficiency anemia, SIRI = systemic inflammation response index.

* Mean (SD), n (unweighted; %).

† Design-based KruskalWallis test; Pearson’s *X*^2^: Rao & Scott adjustment.

### 3.2. Association between SIRI and iron deficiency anemia

The results of the multivariable logistic regression analysis are shown in Table 2. In the crude model (model 1), there was no significant association between lnSIRI and the odds of IDA (OR: 0.91, 95% CI: 0.73–1.14). However, after adjusting for age, gender, and race/ethnicity (model 2), the association became positive and statistically significant (OR: 1.26, 95% CI: 1.02–1.56, *P* = .030). This positive association was strengthened after full covariate adjustment. In the final direct effect model (model 3b), which additionally adjusted for CRP, each 1-unit increase in lnSIRI was associated with 38% higher odds of having IDA (OR: 1.38, 95% CI: 1.11–1.72, *P* = .004).

**Table 2:** Association between SIRI and iron deficiency anemia.

Analysis	Characteristic	Model 1		Model 2		Model 3a (total effect)		Model 3b (direct effect)	
		OR (95% CI)	*P*-value	OR (95% CI)	*P*-value	OR (95% CI)	*P*-value	OR (95% CI)	*P*-value
Continuous analysis	lnSIRI	0.91 (0.73–1.14)	.422	**1.26 (1.02–1.56**)	**.030**	**1.31 (1.05–1.62**)	**.016**	**1.38 (1.11–1.72**)	**.004**
Categorical analysis	SIRI tertiles								
	T1 (lowest)	1.00 (Reference)	–	1.00 (Reference)	–	1.00 (Reference)	–	1.00 (Reference)	–
	T2	0.81 (0.63–1.05)	.106	1.03 (0.79–1.33)	.845	1.05 (0.81–1.37)	.691	1.07 (0.83–1.40)	.584
	T3 (highest)	0.97 (0.75–1.25)	.787	1.40 (1.10–1.79)	.007	1.46 (1.15–1.87)	**.003**	1.55 (1.22–1.97)	**<.001**
	*P* for trend		.783		**.009**		**.003**		**<.001**

Bold values indicate the *P*-value is considered statistically significant.

Model 1: crude, unadjusted. Model 2: adjusted for age, gender, and race. Model 3a (total effect): adjusted for all covariates listed in model 2 plus education, marital status, PIR, BMI, smoking, alcohol, and all comorbidities. Model 3b (direct effect): adjusted for all covariates in model 3a plus C-reactive protein (CRP).

CI = confidence interval, CRP = C-reactive protein, OR = odds ratio, SIRI = systemic inflammation response index.

The analysis of SIRI as a categorical variable (tertiles) yielded consistent results. In the fully adjusted model (model 3b), compared to participants in the lowest SIRI tertile (T1), those in the highest tertile (T3) had a significantly higher odds of IDA (OR: 1.55, 95% CI: 1.22–1.97, *P* < .001). A significant positive trend was observed across the tertiles (*P* for trend < .001).

### 3.3. Dose–response relationship

**Figure 3.**
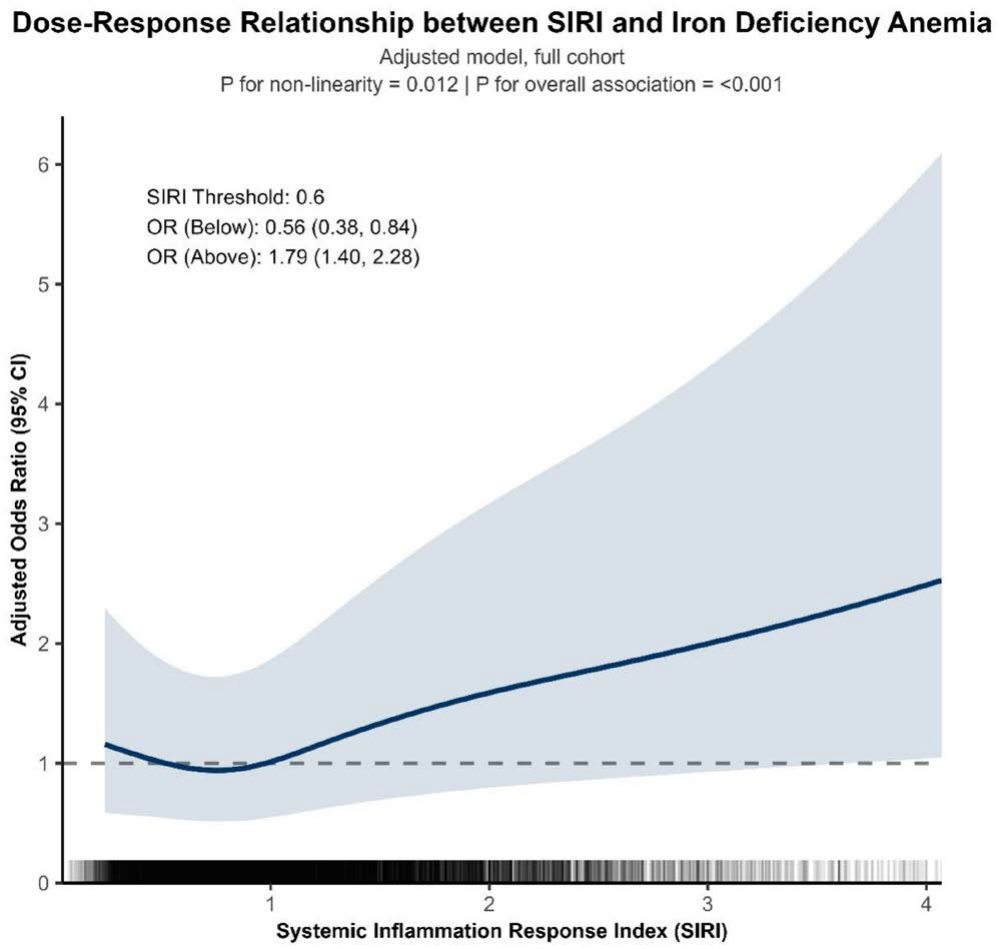
Dose–response relationship between SIRI and iron deficiency anemia. SIRI = systemic inflammation response index.

In fully adjusted restricted cubic spline models, SIRI showed a significant nonlinear association with IDA (*P* for nonlinearity = .012; *P* overall < .001). Risk was flat to slightly decreasing at low SIRI and rose steeply beyond an inflection around SIRI ≈ 0.60; above this threshold, each increment in SIRI corresponded to progressively higher odds of IDA (Fig. 3). Findings were directionally consistent in the sensitivity subset excluding high-grade inflammation (CRP > 10 mg/L), with a nearly identical inflection (~0.61) and significant overall association (*P* < .001; Fig. S1, Supplemental Digital Content, https://links.lww.com/MD/R1910).

### 3.4. Subgroup analyses

Subgroup analyses were performed to assess the consistency of the association between lnSIRI and IDA across various demographic and clinical strata (Fig. 2). Effect modification was significant by sex (*P* for interaction < .001) and diabetes (*P* for interaction < .001). The association between lnSIRI and IDA was stronger in men than in women and stronger in participants with diabetes than in those without. The interaction was not statistically significant for age (*P* = .070), race/ethnicity (*P* = .128), BMI (*P* = .363), or smoking status (*P* = .251). Subgroup-specific estimates are shown in Figure 2 (fully adjusted within each stratum).

### 3.5. Sensitivity and severity analyses

To assess the robustness of our findings, we conducted a sensitivity analysis restricted to 9842 participants with CRP levels ≤ 10 mg/L, thereby excluding individuals with high-grade systemic inflammation. The baseline characteristics of this cohort are presented in Table S1, Supplemental Digital Content, https://links.lww.com/MD/R191). In this subset, the positive association between SIRI and IDA remained strong and statistically significant. In the fully adjusted model, the OR for lnSIRI was 1.35 (95% CI: 1.09–1.67, *P* = .007), and for the highest versus lowest SIRI tertile, the OR was 1.51 (95% CI: 1.17–1.94, *P* = .002; Table S2, Supplemental Digital Content, https://links.lww.com/MD/R191). The nonlinear J-shaped relationship was also confirmed in this sensitivity cohort (*P* for nonlinearity = .003), with a nearly identical inflection point at SIRI ≈ 0.61 (Fig. S1, Supplemental Digital Content, https://links.lww.com/MD/R190).

The analysis stratified by IDA severity revealed that the association was driven primarily by moderate-to-severe cases of IDA (Table 3). After full adjustment (model 3b), lnSIRI was strongly associated with moderate/severe IDA (OR: 1.48, 95% CI: 1.10–2.00, *P* = .011) but showed only a borderline, nonsignificant association with mild IDA (OR: 1.27, 95% CI: 0.98–1.64, *P* = .072). A similar dose-dependent gradient was evident in the tertile analysis, where the *P* for trend was highly significant for moderate/severe IDA (*P* = .004) but not for mild IDA (*P* = .164).

**Table 3:** Association of SIRI with severity of iron deficiency anemia.

IDA severity	Characteristic	Main analysis		Sensitivity analysis (CRP ≤ 10 mg/L)	
		OR (95% CI)	*P* value	OR (95% CI)	*P* value
Mild	lnSIRI				
	Model 1	0.88 (0.68, 1.13)	.314	0.91 (0.69, 1.20)	.504
	Model 2	1.16 (0.91, 1.48)	.235	1.23 (0.94, 1.61)	.133
	Model 3a	1.20 (0.93, 1.55)	.148	1.27 (0.97, 1.68)	.086
	Model 3b	1.27 (0.98, 1.64)	.072	–	–
	SIRI tertiles (model 3a)				
	T1	1.00 (Reference)	–	1.00 (Reference)	–
	T2	1.01 (0.69, 1.47)	.969	0.99 (0.67, 1.46)	.970
	T3	1.26 (0.92, 1.73)	.145	1.36 (0.97, 1.91)	.078
	*P* for trend	–	.164	–	.101
ModSev	lnSIRI				
	Model 1	0.95 (0.68, 1.31)	.739	0.91 (0.67, 1.24)	.561
	Model 2	**1.36 (1.02, 1.82**)	**.039**	**1.35 (1.01, 1.80**)	**.042**
	Model 3a	**1.40 (1.04, 1.89**)	**.028**	**1.37 (1.03, 1.82**)	**.033**
	Model 3b	**1.48 (1.10, 2.00**)	**.011**	–	–
	SIRI tertiles (model 3a)				
	T1	1.00 (Reference)	–	1.00 (Reference)	–
	T2	1.10 (0.78, 1.56)	.576	1.13 (0.77, 1.67)	.530
	T3	**1.68 (1.20, 2.36**)	**.003**	**1.73 (1.21, 2.48**)	**.003**
	*P* for trend	–	**.004**	–	**.004**

Bold values indicate the *P*-value is considered statistically significant.

Methodology: All regression models were weighted using NHANES survey design and results from 5 imputed datasets were pooled using Rubin’s rules.

Severity based on WHO criteria (Hb, g/dL): mild (male 11.0–12.9, female 11.0–11.9), moderate/severe (<11.0).

Models – model 1: crude model. Model 2: adjusted for age, gender, race. Model 3a: fully adjusted model without CRP. Model 3b: fully adjusted model with CRP.

CI = confidence interval, CRP = C-reactive protein, IDA = iron deficiency anemia, NHANES = National Health and Nutrition Examination Survey, N = unweighted sample size, OR = odds ratio, SIRI = systemic inflammation response index.

## 4. Discussion

### 4.1. Summary of principal findings

This large, nationally representative NHANES analysis shows that higher SIRI is independently associated with greater odds of IDA. The relationship is nonlinear with an inflection near SIRI ≈ 0.60 and is particularly pronounced in men and in individuals with diabetes. The association is primarily driven by moderate-to-severe IDA, aligning with biologic plausibility that systemic inflammation (via hepcidin-mediated iron sequestration) contributes to clinically significant anemia rather than marginal hemoglobin changes.^[30]^

### 4.2. Biological plausibility and interpretation of findings

The positive association between SIRI and IDA is biologically plausible and aligns with our current understanding of the inflammation-iron axis. A high SIRI value, reflecting an increased number of pro-inflammatory neutrophils and monocytes relative to regulatory lymphocytes, is indicative of a systemic inflammatory state. This state is known to trigger the production of hepcidin, which in turn orchestrates a systemic reduction in iron availability for erythropoiesis, leading to functional iron deficiency and, eventually, anemia.^[31,32]^ The components of SIRI are directly implicated in this process: monocytes differentiate into macrophages that sequester iron, and both monocytes and neutrophils are potent sources of inflammatory cytokines like IL-6 that drive hepcidin expression.^[33]^

The observed J-shaped dose-response curve provides a more nuanced view than a simple linear model. It suggests that while a basal level of immune activity is homeostatic, there may be a critical threshold of inflammation (approximated by SIRI > 0.6) beyond which the pathophysiological mechanisms linking inflammation to iron dysregulation become dominant, resulting in a sharply increased risk of IDA.^[34]^ This finding could have implications for defining a “high-risk” inflammatory phenotype for anemia development.

A key finding of this study is the robustness of the association in the sensitivity analysis excluding participants with CRP > 10 mg/L. Serum ferritin, a cornerstone for diagnosing iron deficiency, is also an acute-phase reactant, and its levels can be falsely elevated by acute inflammation, masking true iron deficiency.^[35]^ This can introduce a non-differential misclassification bias, potentially diluting the true association between an inflammatory marker and IDA. By removing individuals with high CRP, we mitigated this major source of bias. The fact that the SIRI-IDA association remained strong and significant suggests that the observed relationship is not merely an artifact of acute inflammatory spikes but reflects a more chronic, low-grade inflammatory process that is sufficient to disrupt iron homeostasis.

### 4.3. Comparison with previous literature and contextualization

Our findings are consistent with and extend previous research. A study using the same NHANES dataset found a positive association between the related SII (Platelets × Neutrophils/Lymphocytes) and general anemia. Our study builds upon this by using SIRI, which incorporates monocytes – cells central to iron recycling – and focusing specifically on IDA, a more precise clinical diagnosis.^[33]^ More recently, Guo et al showed that higher SII related to lower ferritin and TBI but higher sTfR and TIBC, with increased odds of iron deficiency and iron-deficiency anemia, and that SII tracked iron deficiency more comprehensively than CRP in US adults; Su et al likewise found a linear SII–anemia association in US children.^[28,29]^ Together these SII-based findings provide population-level context for our SIRI–IDA focus and motivate head-to-head comparisons of SIRI versus SII in future work.

A particularly noteworthy finding was the significant effect modification by gender. The association between SIRI and IDA was substantially stronger in men (OR ≈ 3.5) than in women (OR ≈ 1.3). This disparity can be understood through the differing epidemiology of IDA in these groups. In premenopausal women, IDA is common and is frequently caused by non-inflammatory physiological factors, primarily menstrual blood loss. In this context, while inflammation (as measured by SIRI) may be a contributing factor, its relative importance is diminished among a multitude of other causes. In contrast, IDA in adult men is uncommon and almost always signals an underlying pathology, which is often a chronic inflammatory condition such as inflammatory bowel disease or an occult malignancy leading to chronic blood loss and inflammation. Therefore, in men, a high SIRI is a much stronger indicator of an inflammatory etiology for their anemia, which explains the markedly stronger association observed in our data. A similar logic applies to the stronger association seen in individuals with diabetes, a known chronic inflammatory state.^[36]^

### 4.4. Strengths and limitations

This study has several notable strengths. The use of the NHANES dataset provides a large, diverse, and nationally representative sample, enhancing the generalizability of our findings to the US adult population. The analysis leveraged standardized laboratory measurements and a comprehensive set of covariates, allowing for robust adjustment for potential confounders. Our statistical approach was rigorous, incorporating complex survey design, multiple imputation, sophisticated nonlinear modeling with RCS, and extensive sensitivity and subgroup analyses.

However, several limitations must be acknowledged. First, the cross-sectional design precludes any inference of causality or temporality; we cannot determine whether a high SIRI precedes the development of IDA or is a consequence of it. Second, despite adjusting for a wide range of covariates, the potential for residual confounding by unmeasured factors, such as specific dietary patterns or medication use, remains. Third, the blood markers were measured at a single time point, which may not fully capture an individual’s long-term inflammatory or iron status. Fourth, some comorbidities were ascertained via self-report and may be subject to recall bias. Finally, the diagnosis of IDA relies on established but imperfect laboratory cutoffs, and the definition of iron deficiency in the presence of inflammation is a subject of ongoing debate.

### 4.5. Implications for clinical practice and future research

Despite these limitations, our findings have potential clinical and research implications. SIRI is calculated from a routine, inexpensive complete blood count, making it a universally accessible biomarker.^[37]^ If our findings are validated in prospective studies, SIRI could serve as a valuable, cost-effective tool for risk stratification. It could help clinicians identify patients, particularly men or those with chronic conditions like diabetes, who may have an underlying inflammatory driver for their anemia and could benefit from more targeted diagnostic workups. The identified threshold of SIRI > 0.6 could serve as a preliminary benchmark for heightened clinical suspicion.

Future research should focus on longitudinal cohort studies to establish the temporal sequence and confirm a causal link between elevated SIRI and the incidence of IDA. Mechanistic studies are needed to further elucidate the specific pathways through which the immune cell balance reflected by SIRI modulates hepcidin and iron metabolism. Finally, intervention studies could explore whether therapies aimed at reducing systemic inflammation in patients with high SIRI levels can effectively improve iron status and ameliorate anemia.

## 5. Conclusion

In conclusion, this study provides strong evidence from a large, nationally representative sample that a higher SIRI is independently and nonlinearly associated with an increased prevalence of IDA in US adults. The risk sharply increases above a SIRI threshold of approximately 0.6, with the association being particularly pronounced in men, individuals with diabetes, and for moderate-to-severe disease. Given that SIRI is calculated from routine blood counts, it holds promise as an accessible biomarker to help identify adults at higher risk for inflammation-related iron deficiency. Future prospective studies are needed to validate these findings and to investigate whether modulating systemic inflammation can improve iron homeostasis in high-SIRI populations.

## Author contributions

**Conceptualization:** Yang Li, Tong Li.

**Data curation:** Yang Li, Tong Li.

**Formal analysis:** Yang Li.

**Validation:** Tong Li.

**Writing – original draft:** Yang Li.

**Writing – review & editing:** Tong Li.

## Supplementary Material






