# Supplementary material for: Association between the systemic inflammation response index and iron deficiency anemia in US adults, 2003–2018: A population-based cross-sectional analysis
Source: Medicine (Baltimore). 2026 Jan 23;105(4):e47302. doi: 10.1097/MD.0000000000047302 (PMC12851724; doi:10.1097/MD.0000000000047302)
Supplement: Supplementary file 1 [file medi-105-e47302-s001.docx]

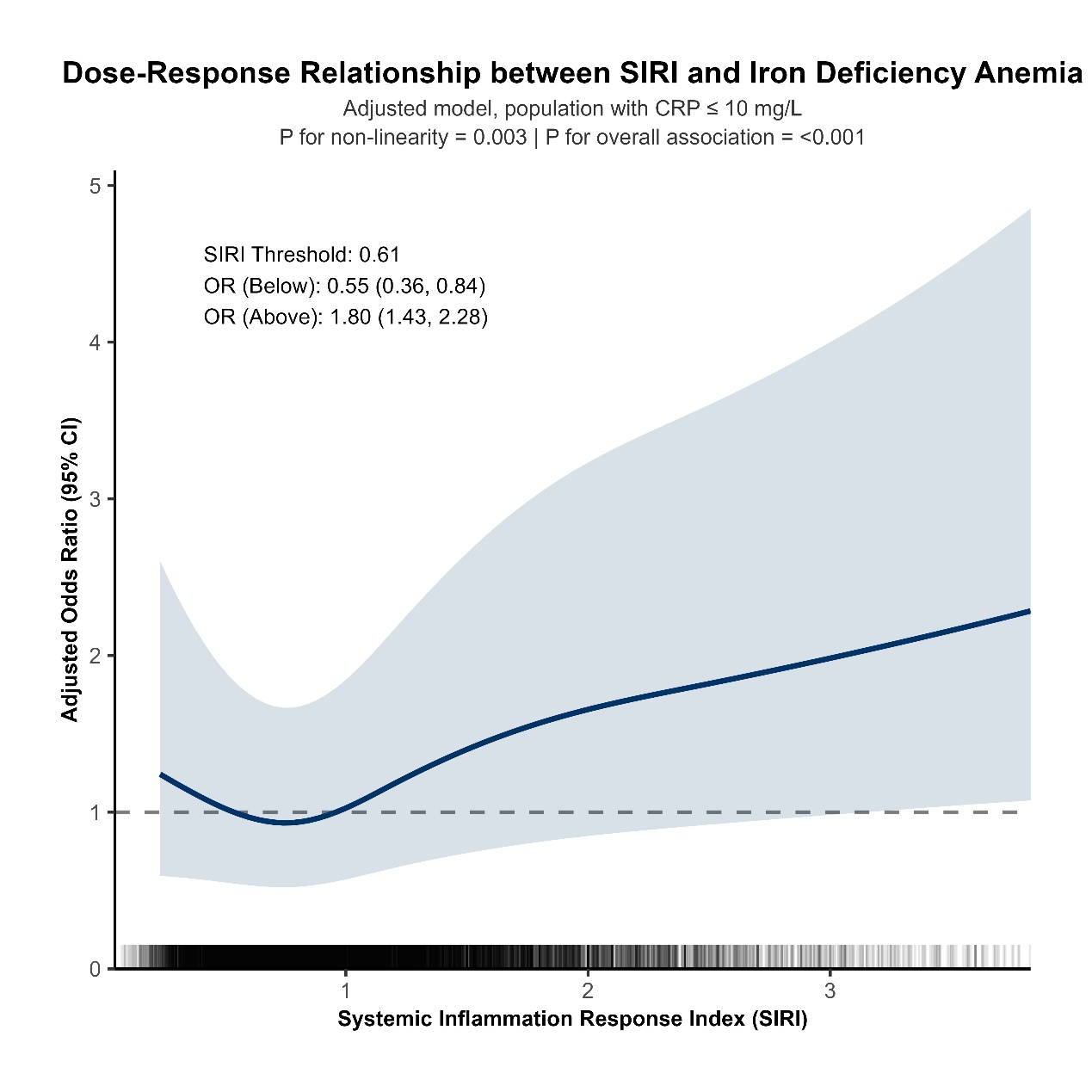


**Supplementary file 1** Figure S1 Dose-Response Relationship between SIRI and Iron Deficiency Anemia. The solid line represents the adjusted odds ratio and the shaded area represents the 95% confidence interval, derived from a restricted cubic spline model. This analysis was conducted in the sensitivity cohort, restricted to participants with CRP ≤ 10 mg/L. The model was adjusted for age, gender, race, education, marital status, PIR, BMI, smoking, alcohol, diabetes, hypertension, CVD, cancer, thyroid disease, and CRP.
