# Supplementary material for: Association between the systemic inflammation response index and iron deficiency anemia in US adults, 2003–2018: A population-based cross-sectional analysis
Source: Medicine (Baltimore). 2026 Jan 23;105(4):e47302. doi: 10.1097/MD.0000000000047302 (PMC12851724; doi:10.1097/MD.0000000000047302)
Supplement: Supplementary file 2 [file medi-105-e47302-s002.docx]

Supplementary file 2 Table S1 Baseline characteristics in the sensitivity cohort (CRP ≤10 mg/L) by IDA status.

| **Characteristic** | **Overall**, N = 9842^1^ | **No IDA**, N = 9223^1^ | **IDA**, N = 619^1^ | **p-value**^2^ |
| --- | --- | --- | --- | --- |
| **Age, years** | 41.3 (15.0) | 41.4 (15.1) | 38.8 (11.0) | 0.042 |
| **Sex** |  |  |  | <0.001 |
| Male | 2,258.0 (22.8%) | 2,225.0 (23.8%) | 33.0 (4.2%) |  |
| Female | 7,584.0 (77.2%) | 6,998.0 (76.2%) | 586.0 (95.8%) |  |
| **Race/Ethnicity** |  |  |  | <0.001 |
| Non-Hispanic White | 3,795.0 (63.8%) | 3,676.0 (65.0%) | 119.0 (40.4%) |  |
| Non-Hispanic Black | 2,083.0 (11.5%) | 1,836.0 (10.5%) | 247.0 (30.4%) |  |
| Hispanic | 2,631.0 (15.7%) | 2,439.0 (15.4%) | 192.0 (21.2%) |  |
| Other | 1,333.0 (9.0%) | 1,272.0 (9.1%) | 61.0 (8.1%) |  |
| **Education** |  |  |  | 0.002 |
| High school | 2,164.0 (23.2%) | 2,018.0 (23.1%) | 146.0 (24.7%) |  |
| Less than high school | 2,068.0 (13.1%) | 1,912.0 (12.8%) | 156.0 (18.8%) |  |
| More than high school | 5,610.0 (63.7%) | 5,293.0 (64.1%) | 317.0 (56.5%) |  |
| **Marital status** |  |  |  | 0.8 |
| Married/Living with partner | 5,836.0 (62.9%) | 5,475.0 (62.9%) | 361.0 (63.4%) |  |
| Never married | 2,199.0 (21.3%) | 2,043.0 (21.2%) | 156.0 (21.8%) |  |
| Divorced/Separated/Widowed | 1,807.0 (15.8%) | 1,705.0 (15.9%) | 102.0 (14.8%) |  |
| **Poverty-to-Income Ratio** | 2.9 (1.6) | 2.9 (1.6) | 2.5 (1.6) | <0.001 |
| **Smoking status** |  |  |  | <0.001 |
| Never | 6,090.0 (59.8%) | 5,643.0 (59.1%) | 447.0 (72.3%) |  |
| Former | 1,714.0 (19.4%) | 1,643.0 (19.8%) | 71.0 (11.2%) |  |
| Current | 2,038.0 (20.9%) | 1,937.0 (21.1%) | 101.0 (16.5%) |  |
| **Alcohol use** |  |  |  | <0.001 |
| Non-drinker | 3,701.0 (30.3%) | 3,424.0 (29.8%) | 277.0 (40.1%) |  |
| Drinker | 6,141.0 (69.7%) | 5,799.0 (70.2%) | 342.0 (59.9%) |  |
| **Body mass index, kg/m²** | 28.2 (6.8) | 28.2 (6.7) | 29.0 (7.6) | 0.14 |
| **Diabetes** |  |  |  | 0.9 |
| Yes | 869.0 (6.5%) | 820.0 (6.5%) | 49.0 (7.1%) |  |
| No | 8,780.0 (91.9%) | 8,220.0 (91.9%) | 560.0 (91.2%) |  |
| Borderline | 187.0 (1.5%) | 177.0 (1.5%) | 10.0 (1.7%) |  |
| **Hypertension** | 6.0 (0.1%) | 6.0 (0.1%) | 0.0 (0.0%) |  |
| Yes |  |  |  | 0.6 |
| No | 2,462.0 (21.6%) | 2,329.0 (21.7%) | 133.0 (20.0%) |  |
| **Cardiovascular disease** | 7,362.0 (78.3%) | 6,876.0 (78.2%) | 486.0 (80.0%) |  |
| **Cancer** | 18.0 (0.1%) | 18.0 (0.1%) | 0.0 (0.0%) |  |
| **Thyroid disease** | 467.0 (3.6%) | 448.0 (3.7%) | 19.0 (2.8%) | 0.4 |
| **White blood cell count, 10³ cells/µL** |  |  |  | 0.015 |
| **C-reactive protein, mg/L** | 677.0 (7.5%) | 652.0 (7.7%) | 25.0 (4.0%) |  |
| **Hemoglobin, g/dL** | 9,158.0 (92.4%) | 8,565.0 (92.2%) | 593.0 (95.9%) |  |
| **Ferritin, ng/mL** | 7.0 (0.1%) | 6.0 (0.1%) | 1.0 (0.1%) |  |
| **Anemia (component)** |  |  |  | 0.7 |
| **Iron deficiency (component)** | 972.0 (10.6%) | 913.0 (10.7%) | 59.0 (10.0%) |  |
| **SIRI** | 8,845.0 (89.2%) | 8,285.0 (89.1%) | 560.0 (90.0%) |  |
| **ln(SIRI)** | 2.0 (0.0%) | 2.0 (0.0%) | 0.0 (0.0%) |  |
| ^1^Mean (SD); n (unweighted) (%); ^2^Design-based KruskalWallis test; Pearson's X^2: Rao & Scott adjustment | | | | |

Supplementary file 3 Table S2 Association between SIRI and IDA in population with CRP ≤ 10 mg/L.

|  |  | **Model 1** | | **Model 2** | | **Model 3a (Total Effect)** | | **Model 3b (Direct Effect)** | |
| --- | --- | --- | --- | --- | --- | --- | --- | --- | --- |
| **Analysis** | **Characteristic** | **OR (95% CI)** | **P-value** | **OR (95% CI)** | **P-value** | **OR (95% CI)** | **P-value** | **OR (95% CI)** | **P-value** |
| **Continuous Analysis** | **lnSIRI** | 0.91 (0.72-1.14) | 0.392 | **1.28 (1.03-1.59)** | **0.024** | **1.31 (1.06-1.62)** | **0.014** | **1.35 (1.09-1.67)** | **0.007** |
| **Categorical Analysis** | **SIRI Tertiles** | | | | | | | | |
|  | **T1 (Lowest)** | 1.00 (Reference) | — | 1.00 (Reference) | — | 1.00 (Reference) | — | 1.00 (Reference) | — |
|  | **T2** | 0.89 (0.70-1.15) | 0.371 | 1.12 (0.87-1.46) | 0.373 | 1.14 (0.87-1.49) | 0.323 | 1.17 (0.89-1.53) | 0.263 |
|  | **T3 (Highest)** | 0.95 (0.73-1.24) | 0.710 | **1.41 (1.09-1.83)** | **0.009** | **1.46 (1.13-1.88)** | **0.004** | **1.51 (1.17-1.94)** | **0.002** |
|  | **P for trend** |  | 0.709 |  | **0.010** |  | **0.005** |  | **0.002** |
| *Model 1: Crude, unadjusted. Model 2: Adjusted for age, gender, and race. Model 3a (Total Effect): Adjusted for all covariates listed in Model 2 plus education, marital status, PIR, BMI, smoking, alcohol, and all comorbidities. Model 3b (Direct Effect): Adjusted for all covariates in Model 3a plus C-reactive protein (CRP).* | | | | | | | | | |
